# A Patient-Initiated Digital COVID-19 Contact Notification Tool (TellYourContacts): Evaluation Study

**DOI:** 10.2196/23843

**Published:** 2021-03-05

**Authors:** Kelechi S Okpara, Jennifer Hecht, Dan Wohlfeiler, Matthew Prior, Jeffrey D Klausner

**Affiliations:** 1 David Geffen School of Medicine University of California, Los Angeles Los Angeles, CA United States; 2 Charles R Drew University of Medicine and Science Los Angeles, CA United States; 3 Building Healthy Online Communities San Francisco, CA United States; 4 San Francisco AIDS Foundation San Francisco, CA United States; 5 National Coalition of STD Directors Washington, DC United States; 6 Department of Preventive Medicine Keck School of Medicine University of Southern California Los Angeles, CA United States

**Keywords:** patient-led digital contact notification, COVID-19, digital contact tracing, contact notification website

## Abstract

**Background:**

Contact notification is a method used to control the spread of infectious disease. In this process, a patient who tests positive for an infectious disease and public health officials work to identify the patient’s close contacts, notify them of their risk of possible exposure to the disease, and provide resources to facilitate the decreased spreading of disease. Contact notification can be done physically in person, via phone call, or digitally through the use of media such as SMS text messages and email. When alerts are made through the latter, it is called digital contact notification.

**Objective:**

For this study, we aim to perform a preliminary evaluation of the use of the TellYourContacts website, a digital contact notification tool for COVID-19 that can be used confidentially and anonymously. We will gather information about the number of website users and message senders, the types of messages sent, and the geographic distribution of senders.

**Methods:**

Patients who chose to get tested for COVID-19 and subsequently tested positive for the disease were alerted of their positive results through Curative Inc (a COVID-19 testing laboratory) and Healthvana (a results disclosure app). Included in the notification was a link to the TellYourContacts website and a message encouraging the person who tested positive for COVID-19 to use the website to alert their close contacts of exposure risk. Over the course of three months, from May 18, 2020, to August 17, 2020, we used Google Analytics and Microsoft Excel to record data on the number of website users and message senders, types of messages sent, and geographic distribution of the senders.

**Results:**

Over the course of three months, 9130 users accessed the website and 1474 unique senders sent a total of 1957 messages, which included 1820 (93%) SMS text messages and 137 (7%) emails. Users sent messages from 40 US states, with the majority of US senders residing in California (49%).

**Conclusions:**

We set out to determine if individuals who test positive for COVID-19 will use the TellYourContacts website to notify their close contacts of COVID-19 exposure risk. Our findings reveal that, during the observation period, each unique sender sent an average of 1.33 messages. The TellYourContacts website offers an additional method that individuals can and will use to notify their close contacts about a recent COVID-19 diagnosis.

## Introduction

The process of contact tracing for COVID-19 varies, depending on the facility. However, the core concept includes first identifying those with an infection. This is done either when an individual tests positive for an infectious disease or if there is belief that there is a high probability that an individual has an infection. Then begins the process of determining if there are any close contacts of the person with COVID-19 that are at risk for contracting the disease. With the assistance of the health care team, patients can be educated on how to identify their close contacts and notify their contacts themselves. Conversely, working with the patient, public health officials and members of the health care team can work together to determine who is a close contact and notify them. The notification of close contacts of their exposure risk can be done in person or via phone call, SMS text message, email, or any additional form of communication. Lastly, the goal of contact tracing and contact notification is to control and decrease disease spread. As a result, when alerting close contacts, resources such as testing, treatments, and educational counselling (including next steps and how to approach the suggested guidelines) can be supplied to facilitate the goal of avoiding the spread of disease [1,2].

Digital contact notification is a form of contact notification where technological media such as emails, SMS text messages, and smartphone apps are used to notify the close contacts of people who tested positive for an infectious disease, such as COVID-19, of their exposure risk [3]. It can allow for greater flexibility within the realm of contact tracing, as opposed to the use of solely in-person or telephone-based contact notification. The use of emails and text messages to notify contacts has been used most frequently in partner notification for HIV and other sexually transmitted diseases (STDs), such as gonorrhea, syphilis, and chlamydia [4-11]. Previous studies on both patient-initiated (where the individual positive for the disease alerts their contacts) and health care provider–initiated digital contact or partner notification have concluded that the use of emails and/or text messages to notify contacts increases the total number of contacts alerted when used in addition to traditional in-person, telephone-based, or written partner notification [5-9]. This method allows for the ability to reach populations that are inaccessible or hard to reach with traditional partner notification [5-7].

However, not all studies have demonstrated a high level of utilization of digital means of notification. In evaluations of inSPOT, a patient-initiated STD partner notification website where those who test positive for an STD can send electronic postcards to their partners, investigators of the project found that <10% of the observed participants sent messages to their partners through the website [12-14]. This could be due, in part, to patients’ preference of notifying their contacts in person [12]. However, more recently, TellYourPartner, an updated version of inSPOT, recorded a high proportion of visitors to messages sent.

We performed a preliminary evaluation of TellYourContacts [15], a patient-initiated COVID-19 contact notification website where users who have tested positive for COVID-19 have the option of sending either text messages or emails to notify their close contacts, to observe the use of the website over a period of three months. As defined by the Centers for Disease Control and Prevention (CDC), a close contact is one who was within 6 feet of the infected person for a cumulative total of at least 15 minutes over a 24-hour period, starting two days before the positive case started experiencing symptoms or two days prior to specimen collection [16,17]. We determined the frequency of website use and the frequency, types, and geographic distribution of messages sent.

## Methods

### TellYourContacts Website

Building Healthy Online Communities (BHOC) and Team Klausner Saving Lives (TKSL) collaborated to create and build the TellYourContacts website, which officially launched on May 13, 2020. TellYourContacts is a rapid adaptation of TellYourPartner, a website on which those positive for an STD can anonymously notify their sex partners [18]. Given the rapid spread of COVID-19 and the serious nature of some symptoms, a number of BHOC partners requested the adaptation of TellYourPartner for COVID-19. Combined with a review of CDC information as well as input from on-the-ground providers, we developed the TellYourContacts website, following quickly evolving best practices on contact notification as they were known at the time.

**Figure 1:**
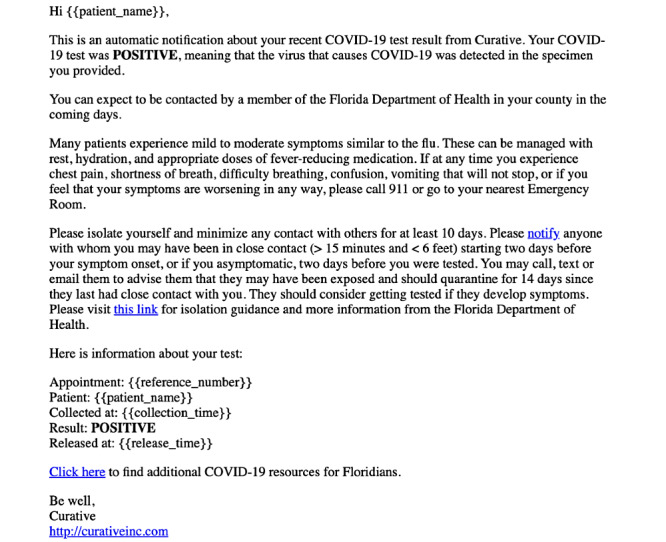
Curative Inc email sent to those with a positive COVID-19 test result, with a "notify" link to the TellYourContacts website (Florida).

Many individuals are first informed about the TellYourContacts website when they are notified of their positive COVID-19 test result by Curative Inc or Healthvana. Within that notification detailing the positive result, a link to the TellYourContacts website is also provided, encouraging patients to notify their close contacts of their risk of contracting COVID-19. Curative Inc is a COVID-19 testing laboratory that supports COVID-19 testing in states throughout the United States and sends patients an email about their COVID-19 test results. If positive, the email contains a link to the TellYourContacts website (Figure 1).

**Figure 2:**
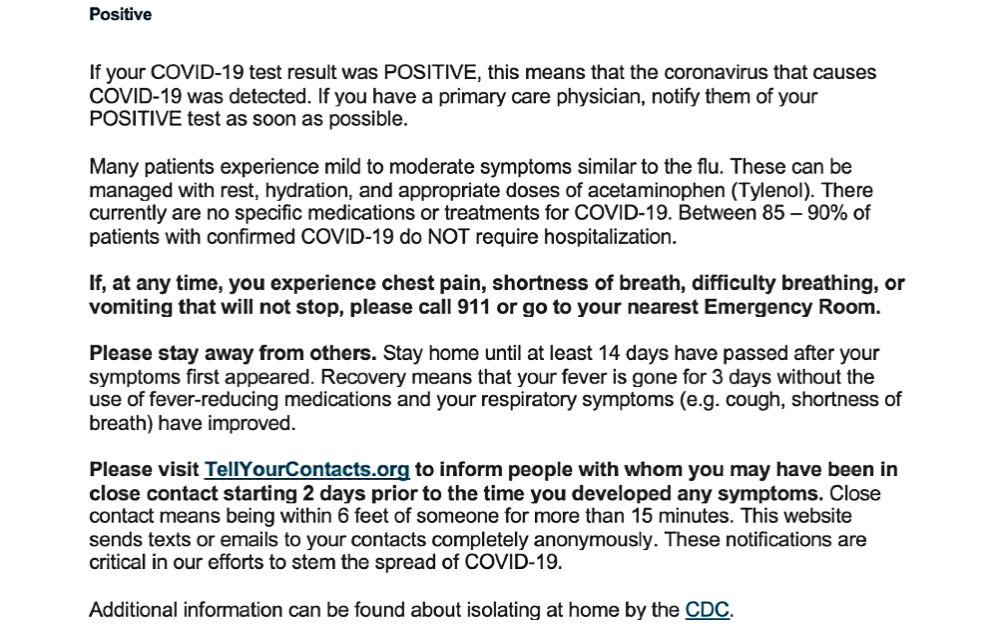
Healthvana message sent to those with a positive COVID-19 test result, with a link to the TellYourContacts website.

Healthvana, a results disclosure app, delivers COVID-19 test results to patients located in California. Within the positive result message, Healthvana sends patients with COVID-19 the TellYourContacts website link [19] (Figure 2).

In addition, the TellYourContacts website was also advertised to members of CDC-funded STD organizations as well as through a press release, social media, and word of mouth.

**Figure 3:**
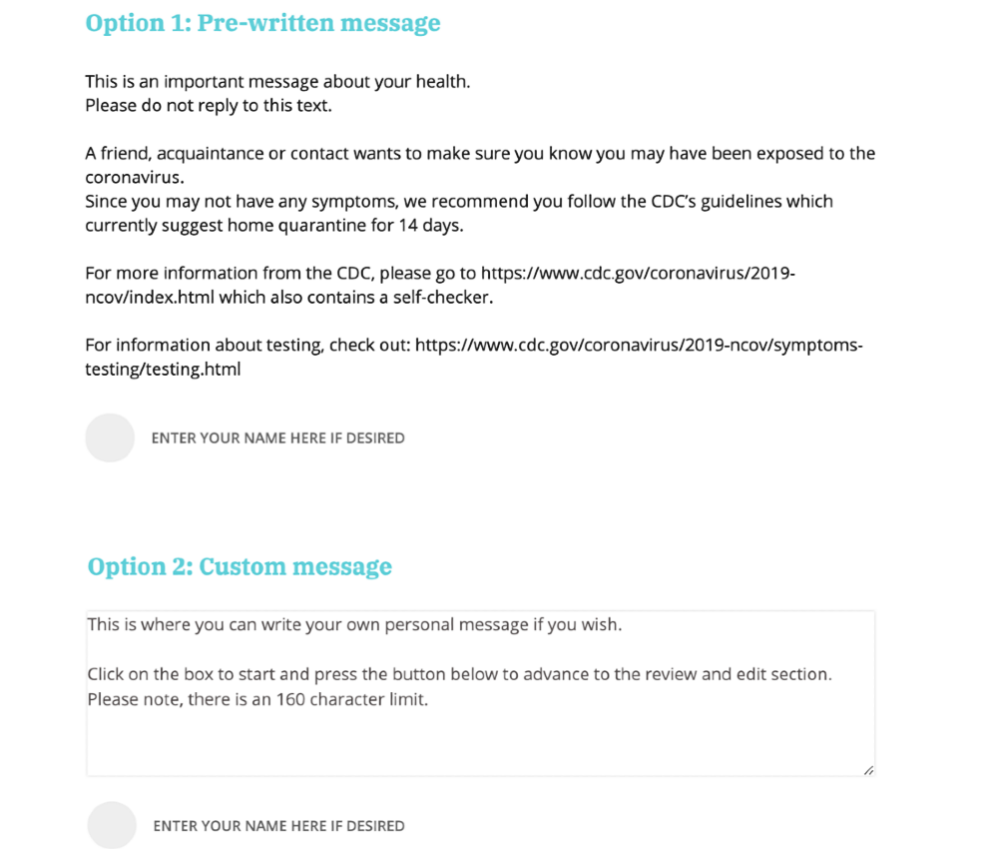
The TellYourContacts website offers prewritten and custom messages for users to send via SMS text message.

**Figure 4:**
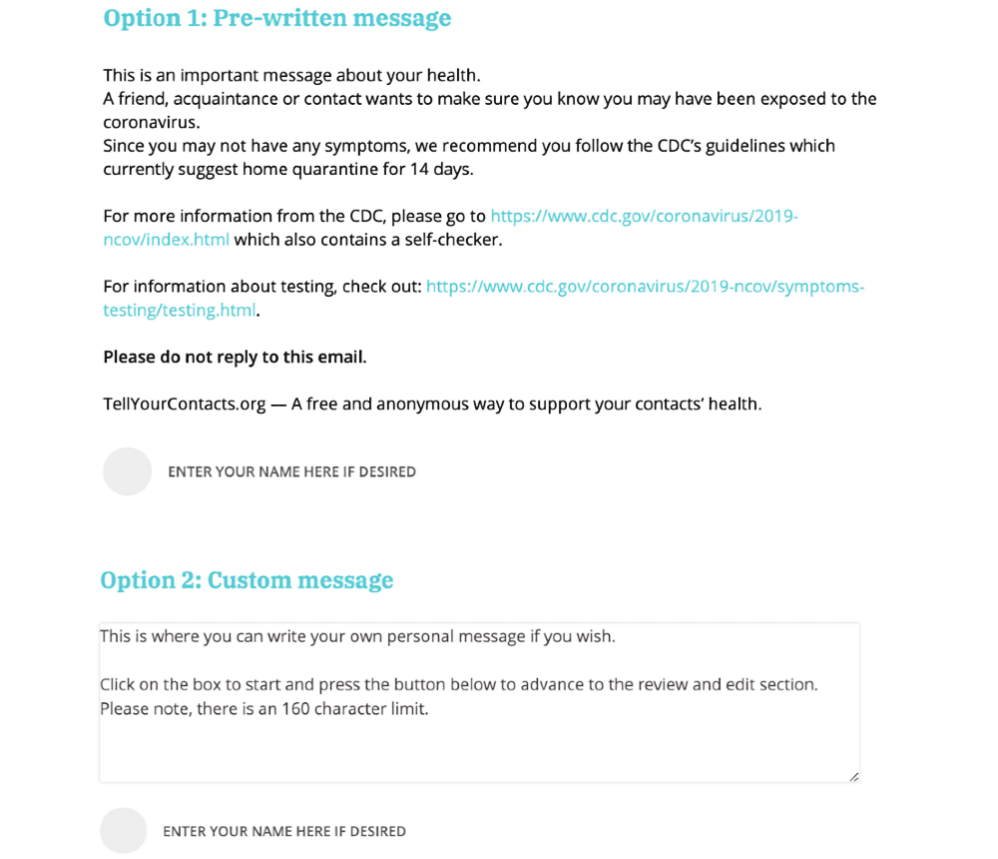
The TellYourContacts website offers prewritten and custom messages for users to send via email.

Once on the TellYourContacts website, users have the option to either send their notification(s) via email or text message to up to 10 close contacts at a time. Additionally, they have the option to send their message(s) anonymously or confidentially, where the user has the option to enter their name. Users also have the option to create their own custom message or to send a prewritten message. The TellYourContacts message alerts the recipient that they may have been exposed to COVID-19 and it is recommended for them to isolate at home as well as seek testing. They are also directed to the CDC’s coronavirus information webpage (Figures 3 and 4).

Lastly, the website provides information about which contacts should be notified and provides direct links to the CDC website for users to learn more about COVID-19 [15,20].

To maintain confidentiality and privacy, TellYourContacts does not record the user’s contact information unless voluntarily provided. As users access the website and input information such as their contacts’ email addresses and phone numbers, their connection and data are encrypted and secured via hypertext transfer protocol by the third-party service provider GoDaddy (GoDaddy Inc). The TellYourContacts website does not use cookies, though its third-party providers may. Additionally, the website neither stores the email addresses or phone numbers of the user’s contacts nor does it link information to an individual. The website also uses services from Mailjet (Mailjet Inc), Twilio (Twilio Inc), and Google Analytics (Google) for email transmission, SMS text message transmission, and collection of aggregate data that includes the total number of users who access the website, the total number and types of messages sent, and the general location of users as determined by internet protocol (IP) address [21]. These providers have their own respective measures of security, which include ISO 27001 Information Security Management compliance and General Data Protection Regulation–compliant email service provider certification [22-24].

### Data Collection and Analysis

We collected data about the total number of emails and messages that were sent by both Curative Inc and Healthvana to notify patients of their positive COVID-19 results. From that, we estimated the number of messages sent to patients observed in our study. Additionally, we collected data regarding TellYourContacts website use including message senders, types of messages sent, and approximate geographic location of senders from Google Analytics. We input the data into Microsoft Excel software (version 16.16.12; Microsoft Corp) to sum the total quantities of the aggregate website data and used descriptive statistics to determine the mean number of messages sent per unique user within the observation period.

## Results

**Figure 5:**
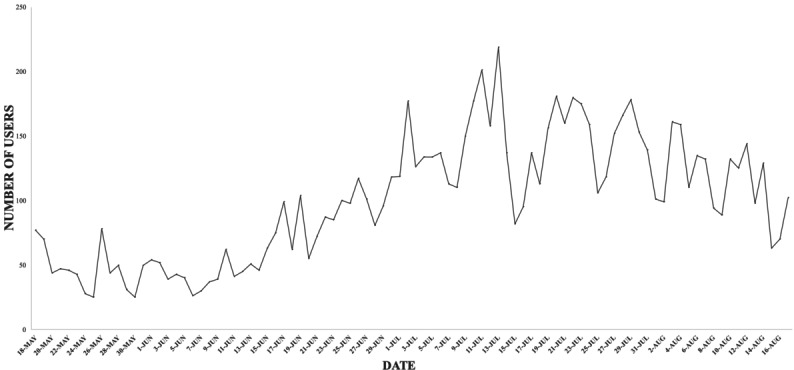
Number of TellYourContacts users per date, from May 18, 2020, to August 17, 2020.

From May 18, 2020, to August 17, 2020, Healthvana reported 125,652 positive COVID-19 test results to patients in California. Additionally, from May 18, 2020, to August 17, 2020, Curative Inc notified 71,866 patients of their positive COVID-19 test results across the United States. During the observation period, from May 18, 2020, until August 17, 2020, a total of 9161 users accessed the TellYourContacts website (Figure 5).

**Figure 6:**
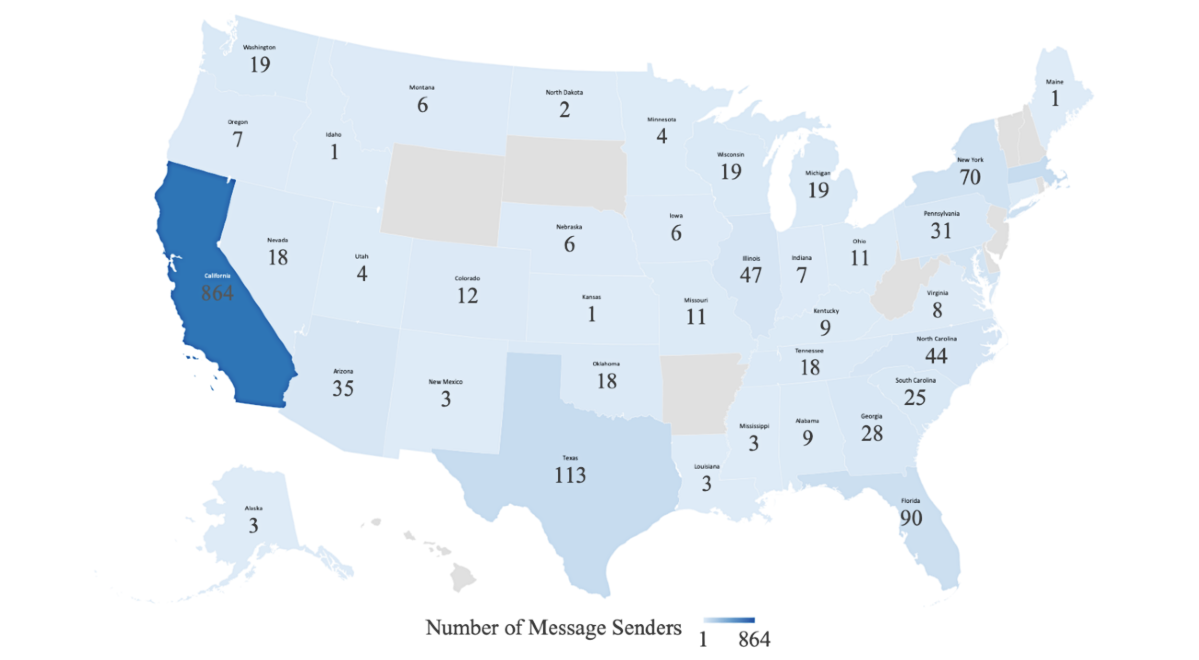
Number of TellYourContacts message senders by state, from May 18, 2020, to August 17, 2020.

Of these users, a total of 1957 sent messages. We found that users chose to send SMS text messages over emails in a 13:1 ratio (1820 [93%] via SMS text messaging and 137 [7%] via email). Messages were sent by users from the following 13 countries: the United States, Canada, Dominican Republic, Mexico, the United Kingdom, South Africa, Japan, Bulgaria, Puerto Rico, French Guiana, the United Arab Emirates, Sweden, Australia, and Morocco. Senders in the United States made up the majority of total senders (97%), representing 40 states. Within the United States, California accounted for the majority of senders (864/1754, 49%). Texas and Massachusetts accounted for the second-most senders, both with 113 (6%) each (Figure 6).

## Discussion

We described the use of a newly created digital notification platform, TellYourContacts, and found that over the course of three months, 9161 users accessed the website and 1474 unique senders sent a total of 1957 messages notifying their close contacts of possible COVID-19 exposure risk; the messages were sent from the majority of states in the United States. Our results demonstrate that individuals will use a digital notification platform to notify their contacts.

Currently, there are various modalities being used for digital COVID-19 contact notification globally, such as smartphone-based contact tracing apps. These apps use either Bluetooth, GPS, or quick response (QR) codes to inform individuals that they may have been exposed to a case. Bluetooth-based contact notification apps measure the signal strength of nearby smartphones to determine the user’s proximity to and duration of an encounter with those infected. GPS-based contact notification apps geolocate users to determine their proximity to those infected. Lastly, QR-based contact notification apps use QR barcodes that are scanned upon one’s entrance to public spaces to monitor the user’s visited locations [3]. In contrast to other forms of COVID-19 contact notification, such as the Apple and Google Exposure Notification System that uses Bluetooth to automatically notify close contacts that have opted in, TellYourContacts assists users in the process of contact notification while maintaining user agency [25]. It gives users the ability to control how they send their messages by providing them with the option to personalize their message or use preformed messages and anonymize themselves. This tool can be especially helpful for users who might not feel comfortable directly telling their contacts or who might not know how to directly tell their contacts about their exposure risk, all while providing their contacts with website links to COVID-19 educational resources. Additionally, TellYourContacts can be used without the need to download and install an app—which would require authorizing additional permissions and continuous updates—on a phone or other electronic device.

Users of TellYourContacts chose to send the majority of messages to their close contacts via text message. This result might possibly be due to the convenience and increasing popularity of text messaging over the past two decades. In 2019, 81% of Americans owned a smartphone, an increase of over 20% compared to five years prior [26]. Geographically, California accounted for the majority of the locations from where messages were sent. This may be because most positive cases observed in our study were tested in California.

This study is not without its limitations. First, our evaluation may have underestimated the number of people who may have received information about the TellYourContacts website because those who were tested at institutions such as nursing homes and homeless shelters were manually excluded from the total observed count. Second, the TellYourContacts website has the potential to be misused, as messages can be sent for unintended purposes. IP addresses are collected by Google Analytics, a third-party provider, to geolocate and provide relevant COVID-19–related information by region. Currently, an anti–IP address spoofing mechanism is in use to filter out false IP addresses associated with bots. Lastly, due to the nature and privacy policies of the website, data regarding the demographic characteristics of each person tested for COVID-19, the percentage of those who sent anonymous messages, and amount of contacts who read the message and took precautions such as isolation, symptom self-checking, and testing, cannot be obtained and analyzed.

With innovations in technology, 90% of Americans use the internet and 81% own a smartphone [26,27]. As a result, digital means such as emailing and text messaging are increasingly used to communicate. TellYourContacts gives individuals who are positive for COVID-19 the ability to initiate contact notification with tools they already use, such as smartphones and computers. After the evaluation of the TellYourContacts website, we found supportive evidence that TellYourContacts is a viable, low-threshold method that people will use to aid in contact notification for COVID-19. Continued social marketing research is needed to further increase its use.

## Abbreviations

BHOC: Building Healthy Online Communities
CDC: Centers for Disease Control and Prevention
IP: internet protocol
NCSD: National Coalition of STD Directors
QR: quick response
STD: sexually transmitted disease
TKSL: Team Klausner Saving Lives
